# Using Assessment for Learning: Multi-Case Studies of Three Chinese University English as a Foreign Language (EFL) Teachers Engaging Students in Learning and Assessment

**DOI:** 10.3389/fpsyg.2021.725132

**Published:** 2021-09-30

**Authors:** Xiaoming (Molly) Wu, Lawrence Jun Zhang, Qian Liu

**Affiliations:** ^1^School of Foreign Studies, Chang'an University, Xi'an, China; ^2^Faculty of Education and Social Work, The University of Auckland, Auckland, New Zealand; ^3^Higher Education Development Centre, The University of Otago, Dunedin, New Zealand

**Keywords:** student engagement, assessment for learning, teacher assessment literacy, goal orientation, trust

## Abstract

Student engagement is an important issue in learning and teaching given its positive effects on students' learning outcomes. Assessment for Learning (AfL), an assessment and pedagogic innovation, if done well, can fully engage students in the learning and assessment process. Adopting a multi-case design, the present study explored how Chinese university English as a Foreign Language (EFL) teachers used AfL to facilitate student engagement in their classrooms and what factors influenced their AfL practices. Three EFL teachers were recruited on a voluntary basis from two universities in Northwest China. Data collected from semi-structed interviews, stimulated recall interviews, and classroom observations suggested that teacher participants demonstrated differed assessment practices, representing Assessment of Learning (AoL), convergent, and divergent AfL, respectively. Three factors: teacher assessment literacy, teachers' beliefs about the relationship between goal orientation and motivation, as well as a trusting relationship between teachers and students, were identified as contributing to teachers' different assessment practices. Our study calls for teacher educators' efforts to equip teachers with necessary assessment-related knowledge and skills, encourage teachers to negotiate learning goals with students, and help teachers establish a trusting environment in their classrooms, if AfL is to be fully embedded in classroom instruction.

## Introduction

Student engagement has no doubt become an important issue in learning and teaching across different education institutions in recent decades (Kahu, 2013; Lim, 2017; Bao et al., 2021; Harris and Leeming, 2021; Rahimi and Zhang, in press). Many studies have shown that student engagement is related to student satisfaction and experience, their learning outcomes and achievements (Pascarella and Terenzini, 2005; Carini et al., 2006; Lei et al., 2018; Teng and Zhang, 2018, 2020; Gan et al., 2021). Despite the importance of engaging students in learning, it is a shared concern that, in practice, engaging students is difficult at almost all educational stages (Skinner and Belmont, 1993; Taylor and Parsons, 2011; Corso et al., 2013; Bundick et al., 2014; Farr-Wharton et al., 2018). Assessment for Learning (AfL), a classroom-based assessment approach and a pedagogical initiative that acknowledges the central role of students, is a possible solution, since research has shown that AfL may well increase student engagement in learning and assessment (e.g., Stiggins, 2010; Swaffield, 2011; Jiang and Zhang, 2021).

In China, AfL has been introduced into university English as a Foreign Language (EFL) classrooms in order to promote student-centered learning, which stresses students' agency, interest, active participation, and responsibilities (Fan et al., 2016). This indicates a paradigm shift in the EFL assessment system: Examinations used to play an important role in Chinese EFL teaching and students' language learning is mainly assessed by in-class examinations, finals, and the high-stakes College English Test (CET), a national large-scale criterion-referenced English test (Liu and Xu, 2017; Zhang et al., 2021). The advocation of AfL in China has challenged the examinations-oriented assessment tradition; it required that teachers no longer treat students as recipients of English language examination results but rather work as assessors themselves and fully participate in the learning and assessment process [MoEC (Ministry of Education of China)., 2017]. Given there is a paucity of empirical studies of AfL in Chinese EFL classrooms (c.f., Wu et al., 2021a,b), our study aims to investigate how Chinese EFL teachers use AfL to engage students in their classrooms and identify key influencing factors underpinning their AfL practices.

The rest of the article first reviews the relevant literature on student engagement, AfL, variations in teachers' AfL practices, and factors contributing to such variations. This is followed by a description of the context in which the present study took place and methods we used to collect and analyze the data. Finally, we present our interpretation of the data and discuss the implications of the study to research and practice.

## Review of the Literature

### Student Engagement and AfL

Engagement is defined as “the interaction between the time, effort and other relevant resources invested by both students and their institutions intended to optimize the student experience and enhance the learning outcomes and development of students and the performance, and reputation of the institution” (Trowler, 2010, p. 3). Previous literature has identified three dimensions of student engagement, namely, that of behavioral, emotional, and cognitive (Fredricks et al., 2005). Behavioral engagement captures student attendance, involvement, and participation; emotional engagement reflects affective outcomes such as interest, enjoyment, and a sense of belonging; cognitive engagement is demonstrated through students' investment in learning that goes beyond the minimum requirements (Bloom, 1956; Fredricks et al., 2005; Trowler, 2010).

One possible solution to engaging students behaviorally, emotionally, and motivationally in classrooms may be the implementation of AfL, which is not only an assessment but also a pedagogic innovation that acknowledges the central role of students in teaching, learning, and assessment (Willms et al., 2009; Bennett, 2011; Taylor and Parsons, 2011; Gardner, 2012; Hawe and Dixon, 2017; Davison, 2019). Assessment for Learning is defined as “part of everyday practice by students, teachers and peers, that seeks, reflects upon and responds to information from dialogue, demonstration, and observation in ways that enhance ongoing learning” (Klenowski, 2009, p. 264). In contrast to Assessment of Learning (AoL) that usually occurs at the end of a learning cycle, serving primarily for the purpose of recording and evaluating students' achievement (Stiggins, 2002; Davison, 2019), AfL requires that teachers collect in-time information about student learning, and use it to inform targeted and specific feedback to guide student learning, and in doing so bring about improvement in students' academic performance and self-regulatory skills (Nicol and Macfarlane-Dick, 2006; Panadero et al., 2018; Wu et al., 2021b). In addition, unlike AoL which enthrones teachers as authorative assessors, AfL requires that teachers engage their students behaviorally, cognitively, and motivationally in classroom activities, and encourage them to work as assessors to make judgment of their own and their peers' learning (Andrade, 2010; Panadero et al., 2016).

Recent developments in AfL have identified five specific strategies, providing clear instruction as to how teachers can implement AfL to engage students in learning and assessment (Wiliam and Thompson, 2008; Wiliam, 2011). The first AfL strategy is concerned with understanding learning goals and criteria for success, which requires teachers not only to involve students in goal setting but also negotiate with, rather than tell, students what they are expected to learn and what desired performance should look like (Sadler, 1989; Carless, 2015). The second AfL strategy is related to collecting evidence of student learning. Teachers are encouraged to fully engage their students using open-ended questions and effective discussions, and in doing so provide students with opportunities to reveal their deep learning (Erickson, 2007; Ruiz-Primo, 2011; Heritage, 2013). Teacher feedback is the third AfL strategy. To encourage student engagement in feedback process, teachers should not deliver unidirectional teacher generated comments to their students, but rather involve students in teacher–student discussions when providing feedback (Carless, 2013; Ajjawi and Boud, 2018; Molloy et al., 2020). The last two strategies are peer- and self-assessment, which require teachers to empower students as assessors, who comment on their own and their peers' work and performance (see e.g., Panadero et al., 2018; Wu, 2020; Wu et al., 2021a,b).

### Variations in Teachers' AfL Practice

Previous studies on AfL have revealed differences in how teachers used AfL to engage students in the learning and assessment process. Torrance and Pryor (1998), drawing on their observations of teachers' classroom assessment practices, identified two distinct ways by which teachers used AfL, which they termed “convergent” and “divergent” assessment respectively. Convergent assessment is the “assessment of the learner by the teacher,” aiming to find out whether a student can do a predetermined task (Torrance and Pryor, 1998, p. 154). It is characterized by relatively closed questioning, strict observation of the curriculum, and teachers dominating the assessment process. Divergent assessment, on the other hand, is concerned with exploring what a learner understands and can do. It involves the use of open tasks, discussions, and questions to collect information to assess student learning. More importantly, students, to a great extent, are encouraged to take responsibility for their own learning, serving as not only recipients of assessment but also providers of assessment information, who are involved in assessment related decision making. In doing so, they are genuinely engaged in assessment and learning in terms of their behavior, cognition, and motivation.

Likewise, Marshall and Drummond (2006) explored how teachers implemented AfL in their classrooms by conducting classroom observations of 27 lessons. They found a great deal of differences existed between these lessons and used “spirit” and “letter” to describe the distinction. Only a small proportion of their teacher participants captured the “spirit” of AfL and genuinely engaged their students in learning and assessment activities in classrooms. The assessment practices of the rest teachers, by contrast, reflected the “letter” of AfL that merely conformed to the prescribed AfL procedures and strategies in a superficial way. Taken together, these studies have indicated that many teachers, in their teaching and assessment practices, may not use AfL in a proper and substantial way to engage students in learning and assessment.

### Teacher Factors Influencing the Implementation of AfL

A variety of teacher factors have been found to influence the implementation of AfL at classroom level (e.g., Heitink et al., 2016; Davison, 2019). Some of these factors are intrapersonal factors, including, among other things, teachers' understanding of the relationship between AfL and AoL (e.g., DeLuca et al., 2012), teachers' agency (Hopfenbeck et al., 2015), and teachers' beliefs about teaching and learning (e.g., Marshall and Drummond, 2006; Earl and Timperley, 2014; Borg, 2015; Gao and Zhang, 2020; Sun and Zhang, 2021; Wang and Zhang, 2021).

One intrapersonal factor that has been repeatedly identified in the literature on AfL is teachers' assessment literacy (Heitink et al., 2016). It, in general, refers to the knowledge and skills regarding assessment (Stiggins, 1995). Teacher assessment literacy encompasses the progressive stages from basic mastery of assessment knowledge and skills to the self-directed awareness of assessment processes and the role of assessors (Xu and Brown, 2016). When it comes to AfL, assessment-literate teachers need to understand, among other things, the roles and responsibilities of teachers and students in the assessment process and what the core AfL strategies are. They also need to have the skills and knowledge to use the core AfL strategies to genuinely engage their students in learning and assessment (Dixon and Hawe, 2018; Dixon et al., 2020). For example, assessment-literate teachers are expected to know how to construct open-ended questions to engage their student in teacher–student dialogue to gain rich information about student learning. Empirical evidence has shown that assessment-literate teachers tended to be more flexible and have more techniques and tools to capture student conceptions and guide their learning when using AfL (Birenbaum et al., 2011; Smith, 2011; Gottheiner and Siegel, 2012), while lack of assessment literacy may lead to teachers' incomplete and superficial implementation of AfL (Zhao et al., 2018).

The literature on AfL has also found an interpersonal factor, a trusting relationship between teachers and students, which is vital to the successful implementation of AfL (Carless, 2013; Panadero, 2016; Dixon and Hawe, 2017; Xu and Carless, 2017). According to Carless (2013), there are two important dimensions of trust: Competence trust and communication trust. Competence trust refers to the trust in a person's ability to perform a task efficiently and effectively. Communication trust incorporates respect, empathy, benevolence, openness, and honesty, which is needed if students are to be fully engaged in assessment activities, especially when they are required to make their learning public in the feedback process. When a trusting relationship exists, students are willing to engage in learning-related tasks (Willis, 2011), take risks (Carless, 2013), and reveal their vulnerability and learning needs (Carless, 2013; Xu and Carless, 2017).

Previous studies have established the important role that teachers play in shaping the effectiveness of AfL and in influencing student engagement, motivation, and success. However, most of the empirical evidence comes from western context. Little is known about how Chinese language teachers use AfL in their classrooms to engage students and what teacher factors may influence their assessment practices (Wu et al., 2021b). Therefore, our exploratory study sets out to fill this research gap, and addresses the following two research questions (RQs):

RQ1: How do teachers implement AfL in Chinese university EFL classrooms to engage their students in learning and assessment? RQ2: What teacher factors influence the way Chinese university EFL teachers use AfL in their classrooms?

## Methods

### Participants

This qualitative exploratory study was part of a larger study which investigated the implementation of AfL in Chinese university English as a Foreign Language (EFL) classrooms. In the larger study, a questionnaire-based survey was conducted first to elicit the frequency of AfL strategies used by teachers in classrooms as well as the values they ascribed to each of these strategies (Wu et al., 2021b). After that, teachers were recruited on a voluntary basis in the follow-up case studies, exploring in depth their classroom assessment practices. Six teachers indicated their willingness to participate by leaving their contact in the questionnaires. In order to explore the different ways in which Chinese EFL teachers use AfL to engage their students in depth, we chose participants for our qualitative study based on three criteria. The first was availability. As data collection in the qualitative phase was estimated to last for approximately 4 months, those who did not fit this time schedule were not considered. The second was teachers' self-reported AfL practices. Teacher participants were chosen to reflect different frequencies of using AfL strategies in their classes based on their responses to the initial questionnaire. Therefore, the third was in consideration of the demographic range. To avoid homogeneity, we selected teacher participants to represent different age range, gender, years of teaching EFL courses, and courses taught. Three were selected out of the six teachers who were willing to participate in the qualitative study and they were given pseudonyms as Nancy, Luke, and Zack, respectively. Zack was an experienced EFL teacher who reported high frequency of adopting AfL and described AfL as having great importance to teaching. Luke and Nancy were two young teachers who reported limited usage of AfL and who failed to realize the values of AfL as indicated in their responses to the questionnaires. The three teachers' demographic information is presented in Table 1 and detailed background information of the teacher participants is reported in the findings section.

**Table 1:** Demographic information of the three teacher participants.

**Name**	**Nancy**	**Luke**	**Zack**
Gender	Female	Male	Male
Age	30	27	47
Teaching experience	6 years	3 years	25 years
Academic credential	Master	Master	Master
Academic rank	Lecturer	Assistant Instructor	Associate Professor
Course taught	College English	College English	Advanced Audio-Visual Speaking
University	B	A	B

### Universities as Data Collection Sites

As indicated in Table 1, the three teacher participants came from two universities in Northwest China, which were both science and technology based universities enjoying similar positions in the QS World University Rankings. In the two universities, all the first- and second-year undergraduates whose major was not English needed to attend the College English course, a 2-year compulsory English course required by the Chinese Ministry of Education. Both two universities followed the national unified College English Syllabus and used the same English textbooks. In addition, they both offered selective English enhancement courses for second-year students who had passed the College English Test band 4 (CET-4 hereafter), a national large-scale English test used to check whether Chinese university EFL students have reached the requirements of the national syllabus in terms of listening, speaking, reading, writing and translation (Gu, 2018). Those students who had not passed this test were required to stay with the College English course. The two universities also adopted a similar school-level assessment policy for the College English course: The students were rated by both their daily performance and their final achievement tests. Students' daily performance, including their attendance, quality of assignments, and engagement in classroom activities, accounted for 30% of the overall assessment, and the final test sores accounted for 70%.

### Data Collection

Prior to data collection, we made initial contact with the participants selected and provided them with participation information sheets (PIS) and consent forms (CF) via e-mail. After they agreed to participate in the study by signing the CFs, each of the teachers was invited to choose one class they taught to be observed. In order to gain a complete picture of how Chinese university EFL teachers implemented AfL and engaged their students in the learning and assessment process, we drew on a variety of methods to collect data, including semi-structured interviews, stimulated recall accounts, and classroom observations, which are explained in the ensuing sections.

#### Semi-Structured Interview

A semi-structured interview following predetermined interview protocols makes comparison of responses easy but also allows for explanation, clarification, and further enquiry of responses (Denzin and Lincoln, 2011). Because of this advantage, we arranged two half-hour semi-structure interviews for each teacher. The first interview with each teacher began with a 10-min discussion about their past EFL learning and teaching experiences to help us know the participant better and to develop a good researcher-participant rapport (Dörnyei, 2007). This was also an opportunity to assess the teacher's understanding of several terms regarding AfL, such as peer- and self-assessment. As some teacher participants were not familiar with these concepts, we briefly described these terms to minimize possible misunderstandings in the subsequent interviews. In the larger study, the pre-written questions (see Appendix A) were informed mainly by the framework of the five core AfL strategies, including goal setting, classroom assessment tasks used, teacher feedback, peer-, and self-assessment opportunities. With the permission of the teacher participants, all the interviews were audiotaped.

#### Classroom Observation

Classroom observation “records behavior as it is happening,” and thus yields direct and first-hand information of the situation (Merriam, 1998). It can also be used in conjunction with other data sources to triangulate findings (Nunan and Bailey, 2009). In our study, classroom observations were used to confirm and complement the data from the participants' self-reported accounts and to understand teachers' practices in a natural setting. The foci of the classroom observations were on how teachers utilized the core AfL strategies to engage their students in the learning and assessment process. Each of our teacher participant's class was observed four times in a non-participatory way to reduce the influence of the observer on the behaviors of the participants (Dörnyei, 2007), with one at the beginning of the term, two in the middle, and one at the end. Each classroom observation lasted for 1 h and a half. There were in total for 24 h classroom observations. Unfortunately, because the teacher participants sometimes refused our request to record their lectures, only part of the classroom observations were videotaped. We hence also made field notes to supplement videotaped data.

#### Stimulated Recall

With some visual and audio reminder, stimulated recall can help elicit more information about participants' mental process during a certain event (Gass and Mackey, 2000). Stimulated recall interviews were used in our study to identify the reasons for teachers' certain behaviors detected in the classroom observations. Each teacher participant was invited to take a 20-min stimulated recall interview 24 h after each classroom observation. Research suggests that data are more reliable if collected sooner after the event (Gass and Mackey, 2000). Therefore, the retrospective data were collected 1 day later, considering the fatigue of the participants and the time needed for us to set up equipment and determine the questions to be asked. For each stimulated recall, the teacher was invited first to watch and reflect on their assessment behaviors during the session that we had observed, and then asked to explain the reasons for their assessment activities.

### Data Analysis

In the preparation stage, for each participant, the semi-structured interviews, stimulated recall accounts, and field notes were organized, formatted, and transcribed for later coding and analysis. Language mistakes, incoherent and incomplete sentences were corrected to make the meaning clear and straightforward. We then read thoroughly the interview data and excluded some irrelated information (e.g., Some teachers gave extensive explanation of the CET-4) from the follow-up data analysis. Meanwhile, we also watched the videotaped classroom observations repeatedly to identify, record, and transcribe the data in relation to teachers' assessment behaviors (e.g., goal setting, classroom assessment tasks, teacher feedback, peer-, and self-assessment) and how they engaged their students in the assessment process.

The data analysis began with holistic coding, namely, assigning a single code to a large unit of data to summarize the overall contents, as advised in Saldaña (2016). Guided by some important AfL literature (Wiliam and Thompson, 2008; Wiliam, 2010) as well as the results of instrument validation in the quantitative phase of the larger study (Wu et al., 2021b), four broad categories were predetermined. These included: *communicating goals to students, elicit information of student learning, teacher feedback, and peer-and self-feedback*. After reading the transcripts repeatedly, we applied these codes to related segments of data and highlighted them with different colors to build a foundation for a more detailed coding. After that, detailed line-by-line coding was conducted under each broad category, and these codes were then subjected to further scrutiny to identify recurring patterns. For example, the analysis of Zack's interview data brought about dozens of codes under the predetermined broad category of *communicating goals to students*. The codes *pass finals and pass high-stakes examinations* were grouped together and were assigned a pattern code of *Learning English to pass examinations*. The codes such as *develop English abilities in working environment* and *improve communicative language abilities* were assigned a pattern code of *learning English to develop abilities* to unify them. These two pattern codes were then grouped together under the theme of *course learning goal*s *advocated*. This theme surfacing from the line-by-line coding, together with other themes generated, seemed to have a different focus from that of the predetermined category *communicating goals to students*; we therefore adjusted the name of the predetermined category into *Course learning goals*.

In the same way, the initial predetermined categories *elicit information of student learning* and *peer-and self-feedback* were renamed as *classroom assessment tasks* and *empowerment of students as assessors*. The data related to teacher feedback were limited and focused mainly on the types of written feedback teachers provided to their students after class, which was beyond the scope of our current study concerned with teachers' use of AfL in classroom settings. The predetermined category *teacher feedback* was hence eliminated. Appendix B provides an example of how the data were analyzed by illustrating the major themes and codes assigned to Zack's data. After analyzed the data of each case, we adopted the replication strategy proposed by Yin (2011) and compared the three cases to identify underlying similarities and/or contradictions based on the major themes generated.

To ensure internal consistency in the coding process, intra-coder agreement was checked. The first author coded five pages of Luke's interview data, and a few days later, she repeated the coding process on the same data. The intra-coder agreement was 88%, within the recommended range of 85–90% for assuming internal consistency (Miles et al., 2015). In addition, peer debriefing was also used to ensure that the first author, who was mainly responsible for the data analysis and interpretation, did not use biased opinions (Dörnyei, 2007). After completing the coding of each case, a Ph.D. candidate from China whose research interest also included AfL was invited to work as a peer debriefer. The first author provided her with samples of raw data, a list of the operational definitions of codes, data display tables for each case and sought her comments on the initial codes assigned as well as the categories and themes generated. Only minor concerns were raised regarding the wording of some codes, which were refined after discussion with the debriefer.

The trustworthiness of our study was also ensured through the quality of translation. Before we wrote up the qualitative findings, the quotes from the interviews and classroom observations excerpts serving as supporting evidence were translated from Chinese to English. This work was done by an EFL teacher in China. A professional translator was then invited to evaluate the translation by checking 20–30% of the translated data for each case. The results of the translation checks indicated overall high quality of translation; only several minor discrepancies occurred which were amended after discussion to reach consensus.

## Findings

The three teacher participants indicated usage of different assessment approaches to engage their students in the learning and assessment process. In this section, we first report the case of Nancy, who mainly relied on examinations and scores to assess and engage her students. We then move on to Luke, who tried to get rid of the influence of examinations in his classrooms yet failed to genuinely embrace AfL. Finally, we present the story of Zack, who were able to capture the spirit of AfL and fully engage students in assessment process.

### Nancy

Nancy was a young teacher in her early-thirties, who has been working as a lecturer at a university in Northwest China for more than 6 years. When invited to this study, she was teaching College English to three classes. An important feature that distinguished Nancy from the other two teacher participants was her success in examinations. Nancy ranked in the top 10% among over 300,000 test-takers in her province in the University Entrance Examination to Higher Education. she was hence admitted to one of the most prestigious universities in China, majoring in English. Four years later, owing to her outstanding performance in the Graduate School Entrance Examination (GSEE), she entered the postgraduate school of the same university to pursue a master's degree, specializing in linguistics. When she participated in this study, she had just passed the Doctoral Candidate Entrance Examination and had been successfully admitted to a doctoral program in a top university in China. Nancy merely reported a 2-day pre-service training experience in the first month of her teaching career, which did not include any substantial assessment-related courses. She also shared that she never heard of AfL and knew almost nothing about this concept.

Nancy described her EFL teaching experience as “frustrating” (Nancy Int1) (Nancy Int1 means the data were from Nancy in her first interview). She shared that she felt “desperate” every time she started a lesson: “I had to hold my temper and take a deep breath before I entered the classroom” (Nancy Int1). According to Nancy, her extremely negative feelings resulted mainly from the “indifference” of her students, who she perceived as having low English proficiency, limited interest in, and a negative attitude toward learning English. She felt it “extremely difficult” to involve her students in her class, as she commented:

I have given my students opportunities [to take part in class activates]. What can I do if they do not take it? For those students who do not care about their learning, no matter how hard I try, it makes no difference. It was a complete waste of time and energy. (Nancy Int1)

#### Course Learning Goals: Learning English to Pass Examinations

According to Nancy, most of the students she taught “had low interest in English” and “only cared about passing examinations” (Nancy Int1). Nancy shared what she had observed:

Many of my students hate English. Most of the time, they either sleep or play with their mobile phones [during lessons]. But whenever you talk about examinations, they become fully focused …. If they do not see any connection between what you are teaching and the examinations, they will consider it as a waste of time to learn it. (Nancy Int1)

Therefore, Nancy felt “compelled” to cater to her students and hence related the College English course to examinations, which she believed was “the only way to stimulate [her] students' enthusiasm and sustain their efforts to learn English” (Nancy Int1). For this reason, she stressed repeatedly in her classes how learning the College English course could help her students pass final examinations as well as the two high-stakes language tests, the CET-4 and CET-6. For instance, in the second session observed, Nancy and her students were working on reading passage A of Unit 2, which revolved around the impact of greenhouse gas emission on the marine environment. Nancy explained to her students how learning this passage could help them in the coming final examination:

It is possible you will need to write an essay about environmental protection in the final examination. Environmental protection is a hot topic. If you do not learn the passage well … If you know nothing about the impact of greenhouse gas, how can you write a good essay and pass the final examination? (Nancy Obs2) (Nancy Obs2 means the data come from the second classroom observation of Nancy's class)

#### Classroom Assessment Tasks: Examinations and Bonus Scores

Frequent examinations were observed in Nancy's class. At the beginning of almost every session, Nancy carried out a word dictation to check her students' spelling of the words they had learnt in the previous session. In addition, Nancy allocated 30 min for a written test once she finished a unit, focusing on the linguistic knowledge in the unit. In doing this, Nancy expected to help her student “find out the areas they needed to pay more attention to” (Nancy SR2) (Nancy SR2 means this quotation comes from Nancy in her second stimulated recall interview) and encourage students to “work hard in their English learning…because low examination scores is a salutary reminder of [their insufficient efforts in learning the English course]” (Nancy SR3).

In her instruction, Nancy also used multiple-choice questions, filling-in-the blank questions, sentence translation to identify words, and grammatical points with which her students had problems. Nancy was not satisfied with her students' reactions to her questions as only a couple of students responded actively, while a large percentage of students “did not raise their hands for the whole term” (Nancy Int2). Sometimes, even when Nancy addressed her questions directly to these quiet students, a few “merely stood up, keeping silent or uttering *I do not know*” (Nancy In2). Nancy, therefore, employed what she called a “system of rewards and penalties” to promote her students' engagement in questioning. Nancy divided her students into eight groups, and when a student provided a correct answer to Nancy's question, they could earn their group one point. Each member of the group with the highest points was awarded “bonus scores” added to his/her final scores of the term, while each member of the group with the lowest points was required to write an essay as a punishment. Nancy considered “giving bonus scores” to be “the most effective tool” to engage her students, especially for students who had not realized the value of these activities in enhancing their language learning:

Most of my students do not take it (answering questions) as a learning opportunity …. I guess they even think that you are annoying if you keep on asking questions. But because their performance will be scored and added to the final scores, they have to be active. (Nancy SR2)

Nancy admitted that she seldom asked open questions, or organized discussions in her class, which she attributed to her students' “poor language ability and thinking ability” (Nancy Int1). She gave an example of an unsuccessful attempt to involve her students in a discussion related to the differences between Chinese and American culture. First, she asked her students to brainstorm the areas where the differences might exist; her students were able to give simple answers, such as food, fashion, and education. However, when Nancy further asked them to describe the differences in detail and to explain how these differences came into being, her students “were baffled,” and “all kept silent” (Nancy Int1). Nancy then reduced the difficulty of the question by permitting her students to respond in Chinese, but the students still “wore a look of incomprehension” (Nancy Int1). Nancy concluded that her students “neither had enough vocabulary to express themselves nor had the ability to develop their ideas and analyze the problems” (Nancy Int1). Nancy, therefore, decided that it was “safer to ask questions that student could handle” and chose not to challenge her students to “avoid embarrassment” (Nancy Int1).

#### Empowerment of Students as Assessors: Peer- and Self-Assessment Giving Way to Examination Preparation

In all the four sessions observed, Nancy's students were not given any opportunity to assess, or comment on, the work of their peers or their own. This is confirmed by the interview data, as Nancy described that she “did not provide many opportunities for [her] students to do peer- or self- assessment” (Nancy Int2).

When asked about the reasons for the rare occurrence of peer-and self-assessment in her class, Nancy responded that peer- and self-assessment would “take up a lot of teaching time” (Nancy Int2). Nancy considered it as her “first duty to finish the unified teaching plan” in the limited teaching hours because the final examination was designed based on this. Nancy worried that “[her] students would complain if [she] missed the content to be tested in the final examination” (Nancy Int2). Peer- and self- assessment, hence, needed to give way to the content to be tested in the final examinations in her class.

Nancy also shared that peer- and self-assessment “[would] not work out” in her current class (Nancy Int2). She doubted her students' ability in giving quality peer-feedback because of their low English proficiency. As she explained: “some students are bad in English. It is not easy for them to make themselves understood, and how could it possible for them to give feedback?” (Nancy Int2). Furthermore, Nancy was quite certain that her students could not be fully engaged in self-and peer-assessment based on her previous teaching experience. Nancy once experimented with group study and self-study in her class, but most of her student “either sat around, doing nothing or talked about celebrity gossips” owing to their “lack of self-control” and “low motivation and interest in learning English” (Nancy Int2).

### Luke

Luke was in his late 20th and had been working as an assistant instructor at a university in Northwest China for 3 years. When he participated in the current study, he was teaching College English to three classes. What attracted us to Luke's story most was his EFL learning experiences. Luke repeatedly stressed the great influence his English teachers had on his EFL learning. His EFL teacher in junior middle school “only taught [him] what would be tested” and “always pulled a long face when [he] got low scores,” which was so “discouraging” that he wanted to “give up on English” (Luke Int1). His EFL teacher in senior middle school, in contrast, “unlike most of [his] other English teachers who always stressed test scores,” drew Luke's attention to the “improvement of pronunciation and English speaking and listening abilities” (Luke Int1). Because of this teacher, Luke's interest in English increased greatly.

Luke reported that he did not receive much pre-service or in-service training regarding assessment. There were no assessment-related courses when he was at university; he also complained about the insufficient support provided by the university where he worked. As Luke shared:

I haven't received any training [about assessment]. The university only requires publications from teachers or issues administrative regulations and requirements to manage teachers. It pays little attention to teaching. Although there is a teacher development center, I have no idea what it is for. (Luke Int2)

#### Course Learning Goals: Learning English to Develop Abilities

Luke was fully aware of the negative impact of examinations on his students. Through observations and informal talks with his students, he found out that a large proportion of his students lacked confidence, interest, and motivation in learning English, which he attributed to their unsatisfying performance in the previous examinations in middle school. Luke conveyed how he felt about his students:

What almost all my students have in common is that they are not interested in English at all. The biggest reason is that their test results were not good in middle school. They feel unfulfilled, so they naturally do not want to learn English anymore. (Luke Int1)

Although Luke was aware that most of his students took passing CET-4, CET-6 and final examinations as “their ultimate goal of learning the College English course,” he expected his students to focus more on the “improvement of their listening, speaking, and genuine language communicative abilities,” and to “utilize English as a tool to know about this world” (Luke Int1). Luke shared that from his EFL learning experiences, he had realized: “stressing test scores will not help students… If teachers make their students focus on the improvement of their language abilities, students will be more interested in English, and will naturally have good performance in examinations” (Luke Int1).

Luke was confident that focusing his students on the improvement of their abilities could “increase [his] students' interest in English learning” and “activate them both in and out of class” (Luke Int1). Luke related how his previous students had changed their behaviors after their focus shifted in the College English course from test scores to the improvement of authentic English:

They started to listen to my lecture attentively and had more eye contact with me…More students opened their month and followed me to do practice… Some students told me they started to listen to English songs and watch English movies on their own initiatives… I felt it was helpful at least to some of my students. (Luke Int 1)

The observation data also provided clear evidence that Luke constantly stressed the importance of genuine English communicative capabilities in his class. For example, in the first session observed, Luke explained to his students that these abilities rather than high examination scores were the key to career success in an international company: “The most important thing is you can convince your boss and express your own opinions articulately and confidently in English. Nobody cares about your scores” (Luke Obs1).

#### Classroom Assessment Tasks: Closed Questions

Luke shared that he “was unwilling to test [his] students” because he was afraid that it would “make them feel stressed” and he wanted them to “focus on the improvement of their English communicative abilities” (Luke Int1). Luke said “I always tell my students I won't prepare them for examinatons …. I also do not want to give them a lot of examinatons in my class” (Luke Int1). The observation data also confirmed what Luke said in the interview, with no examinatons observed in all the four sessions.

The classroom observation data indicated that Luke asked questions to assess his students' mastery of knowledge, and most of them were closed in nature. The following classroom observation extract provides a typical example, where Luke asked his students a series of questions concerning the word “backpack” after explaining the rule of pronunciation change of vowels in stressed and unstressed syllables. The dialogue is as follows:

Luke: “How many syllables? Tell me.”

Students gave no response.

Luke: “two syllables, right?”

Students: “Yes.”

Luke: “How many vowels in this word?”

Luke and Students: “Two.”

Luke: “Are the two vowels the same?”

Students: “Yes.”

Luke: “Do we pronounce them in the same way? “

Students: “No.”

Luke: “What is the difference?”

One student: “One is a stressed syllable. The other one is an unstressed syllable.”

Luke: “Which one is the stressed syllable?”

Students: “The first one.”

Luke: “How to pronounce this vowel in this stressed syllable?”

All students kept silent.

Luke was also observed to invite his students to participate in some other class activities. For instance, at the beginning of most sessions, he allocated 10–15 min for a student to do an oral presentation (Luke Obs 1, 2, and 4), and he also required his students to work in groups or pairs to provide captions for English movie clips (Luke Obs 2). These activities, however, according to Luke, were only “used occasionally” to “liven up the classroom atmosphere.”

#### Empowerment of Students as Assessors: Lacking Understanding of Students' Active Roles in Assessment

Luke asserted that most of the assessment practices in his class were “teacher-led” (Luke Int2). The observation data only indicated a couple of superficial peers- and self- assessment practices. For example, Luke was observed to ask his students to compare their pronunciation of some words containing the consonant /v/ with a video clip, in which a native speaker modeled how to pronounce these words (Luke Obs2). He also required his students to check each other's spellings after they finished an exercise in the textbook (Luke Obs3).

When asked about his understanding of peer- and self-assessment, Luke said he had “never heard of this concept” (Luke Int2). After being given detailed explanation and some examples of these student-led assessment practices, Luke reflected on his assessment behaviors, attributing his rare use of peer- and self- assessment to his deeply held belief in what he called “the hierarchical difference between the teacher and his students” (Luke Int2). Luke explained:

I felt like teachers was there [Luke pointed to the ceiling], and the students were here [Luke pointed to the floor]. Because it [the relationship of the teacher and his students] was like this.… I thought teachers should definitely be the assessor…. I never thought of asking my students to do assessment by themselves. In China, teachers and students have got used to it [teacher being the assessor] (Luke Int2).

Luke further commented that it was an “inspiring” idea to place students in the center of the assessment process and expressed a strong willingness to experiment this AfL strategy in his future teaching:

It is a pity I knew nothing about it [peer- and self-assessment]. It might be a good method. I will definitely use it, and I think my students will have a strong sense of accomplishments and their interest in learning English will grow. (Luke Int2)

### Zack

Zack, who had been working as a university EFL teacher for 25 years, was the most experienced teacher among the three teacher participants. When he was recruited for this study, he was in his mid-forties and worked as an associate professor, being responsible for a College English Enrichment course, Advanced Audio-visual and Speaking. According to Zack, his research experience in the field of second language teaching and learning made him “open-minded about new concepts” (Zack Int 1). For example, Zack was the first in his department to experiment with project-based teaching, a pedagogic approach aiming to help students acquire deeper knowledge through authentic projects. Zack was also responsible for a teaching reform program funded by the province to help young EFL teachers gain skills and expertise to carry out this pedagogy in their classrooms.

Zack was the only teacher participant who “[had] heard of AfL” (Zack Int1). Zack shared that he had attended a seminar concerning AfL when selected by the university to participate in an EFL teacher training program in England. Zack was “amazed at” the research findings presented in a seminar on the effectiveness of AfL in promoting student learning, and was inspired to read further relevant literature to “find out how [he could] apply this idea in [his]classroom” (Zack Int1). Zack valued AfL and believed experimenting with this new assessment approach could help him create the “ideal class,” which he described using the following metaphor:

I hope my students can play a leading role in my class. They are actors and actresses on the stage, displaying their talents and abilities in English. I will take a supporting role, acting as a director off the stage, merely organizing some class activities and solving their problems when necessary. (Zack Int1)

#### Course Learning Goals: Learning English to Pass Examinations and to Develop Abilities

Zack believed that “[his] students differed greatly” in terms of “their motivations to learn English” (Zack Int1). Therefore, he advocated different course learning goals based on students' individual situations. According to Zack, 10% students in his class were “study masters,” 20% were “study slackers,” and the rest were middle-level students (Zack Int1). The top 10% students, as described by Zack: “still [had] great enthusiasm for learning English and high expectations for themselves after passing the CET-4.” Moreover, “they also [had] good study methods and [knew] how to make use of different learning resources” (Zack Int1). Since these students were self-directed and had a strong motivation to learn English, Zack expected them to focus on the “improvement of their English language abilities” when learning the Advanced Audio-visual and Speaking course, which was “beneficial for their future career development” (Zack Int1).

As for the rest of his students, Zack felt they “[had] reduced their efforts in learning English after passing the CET-4.” Zack deemed it necessary to encourage these students to sign up for the CET-6 or other Language tests such as the TOEIC or the IELTS to maintain their efforts in learning English: “you cannot emphasize the importance of examinations to them too much. These students only want to spend time on English when they need to pass examinations.” (Zack Int1) However, Zack believed that high-stakes testing “[could] not work as a sustained motivation,” and therefore, it was of ultimate importance for teachers to “gradually draw students' attention to the development of authentic language use capabilities in the examination preparation process.” (Zack Int1). The classroom observation data also provided evidence that Zack advocated flexible course learning goals. Zack was observed to provide suggestions to students as to what examinations they could prepare for as well as what learning resources students could use for test preparation (Zack Obs 2). Meanwhile, he was also observed to focus his students on the improvement of their abilities by providing role models, telling them that some of his previous students had become successful in their careers owing to English communicative capabilities (Zack Obs3).

#### Classroom Assessment Tasks: Open Questions and Dialogue

According to Zack, the assessment method he used the most in his classroom was “asking questions” (Zack Int1). For each lesson, Zack would “deliberately prepare several key questions” which were “related to the teaching content” and “aligned with students' average English level” (Zack Int1). Most of the questions he asked were open-ended and speculative in nature. For example, he raised questions requiring students to compare [e.g. “Does criticism do more harm than good to people?” (Zack Obs1)], to evaluate [e.g. “Is it a good idea to control population growth?” (Zack Obs4)], to analyze [e.g. “What are the main reasons for the air pollution in China?” (Zack Obs2)], and to solve [e.g., “How to solve plastic pollution?” (Zack, Obs2)].

Zack sometimes extended his planned key questions by asking improvised follow-up questions in English. He sequenced these questions strategically to facilitate teacher–student dialogues and hence making his students' thinking explicit, which can be exemplified by the following excerpt from the third classroom observation. In that session, Zack and his students were working on unit three, *Work to Live or Live to Work*. After playing a videotaped interview of two men (Mr. Smith and Mr. Brown) talking about their work and life, Zack directed a pre-set question (“What is the value of work?”) to a girl seated in the first row in order to “find out what understanding of the interview she had gained” and “what she would express in English on this topic” (Zack SR3). The dialogue is presented as follows:

Zack: “In your opinion, what is the value of work?”

Student: “The value of work? … I do not know how to say…”

Zack: Ok. “Let me change a question. What is Mr. Smith's attitude toward his work?”

Student: “I think he only works for money.”

Zack: “Then what is Mr. Brown's attitude toward his work?”

Student: “He always chooses the work he likes.”

Zack: “Very good. If you must make a choice between two positions, one is well-paid but boring, while the other one makes you happy, yet the salary is low, which one will you take?”

Student: “I think I will choose the first one.”

Zack: “Why?”

Student: “Because if I have money, I can have a good life. I can live comfortably.”

Zack: “What else?”

Student: “I have family responsibilities. I am the only child in my family. My parents need me when they become old.”

Zack: “Do you mean you need money to support your family?”

Student: “Yes. If they [are] sick, I need a work with high salary.”

Zack: “All right. Now turn back to the first question: what is the value of work?”

Student: “I think the value of work is to bring a better life to your family.”

Zack shared that he “prefer[red] open questions” because they challenged his students to “expand on their answers rather than merely saying a couple of words,” and hence gave him rich information about what his students knew, understood and could do (Zack Int1). When asking questions, Zack would walk among his students, addressing a predetermined question to four to five students he chose randomly, because he aimed to “make sure most students can get the opportunity to answer [his] questions” (Zack SR2). Zack attached great value to this type of “teacher–student interaction” in English language learning, which he believed could “force [his] students to speak more” and “help them build their confidence in answering [his] questions” (Zack SR3).

#### Empowerment of Students as Assessors: Successful Experience of Implementation of Peer- and Self-Assessment

Zack shared with us in his interviews how he carried out frequent peer- and self-assessment in one of his previous English writing classes. He divided his students into 5 groups and appointed the one with the highest English level to be the leader of each group. Every time students finished their writing, they were first required to work in pairs to comment on each other's draft with regard to the content, organization, grammar accuracy, and lexical range based on the writing rubric used in the CET-4. Their work was then submitted to the group leader, who would provide further comments based on the same criteria. After that, students' work would be returned for revision and they were required to conduct self-reflection on how they could use the feedback from their peers to improve their work. During this process, students were encouraged to discuss the feedback with the feedback givers. Finally, the second draft, as well as the peer-feedback and students' written self-reflection, went to Zack, who gave feedback not only on students' work but also on the comments generated by their peers, for final review. During breaks between sessions, Zack made himself available for students to discuss with him their writing, and the feedback they received, as well as any problems in giving and interpreting feedback (Zack Int2).

The self-and peer-assessment practices lasted for a whole term and was a “big success” (Zack Int2). According to Zack, the writing ability of most of his students greatly improved, which was confirmed by the high scores they achieved in the writing module of the CET-4. Students' assessment skills and knowledge also increased, and their understanding of the writing rubric deepened. At the beginning of the term, students could only “find out grammatical or spelling mistakes,” but gradually they could also “provide constructive comments on the structure of writing and the development of ideas” (Zack Int2). Furthermore, students' confidence was also enhanced. As reported by Zack, one of his students, who told him that they found that English writing was not as difficult as they had thought, started to write a diary and novels in English.

Both the observation and interview data, however, indicated that Zack provided almost no opportunity for his current students to work as assessors. According to Zack, it was because his current students had unsatisfactory English abilities and assessment expertise, which, he assumed, would exacerbate his workload. Zack commented through recollecting his previous experience of experimenting peer- and self-assessment:

The feedback given by my students had grammatical mistakes and sentences that did not make sense, so I spent a lot of time correcting their feedback…I can image how tiring it will be if I do it [peer- and self- assessment] in my current class (Zack, Int 2).

Besides, Zack believed “the time [to carry out peer-and self-assessment] [was] not yet ripe” in his current class because the class atmosphere was “not lively enough” and “the students [had] not been fully mobilized” (Zack Int2), Zack, from his experience, had realized that building up a trusting class atmosphere was conducive to student-centered assessment practices, as he put it:

If the teacher has emotional communications with his students and creates opportunities for students to make friends with each other, students will gradually feel relaxed and the classroom atmosphere will be active. Then the teacher should encourage his students to take the initiative (in the assessment process) (Zack Int2).

## Discussion

It has long been acknowledged that engaging students in learning behaviorally, cognitively, and motivationally is a prerequisite for students' academic success (Carini et al., 2006; Teng and Zhang, 2018, 2020; Harris and Leeming, 2021; Wu et al., 2021a). AfL, a classroom assessment approach which brings students to the forefront of learning and assessment, has provided a specific prescription as to how teachers can engage their students by using the five core AfL strategies (i.e., goal communication, effective in-class assessment tasks, teacher feedback, peer-, and self-assessment) (Wiliam and Thompson, 2008; Swaffield, 2011). Our study explored in depth how teachers used AfL in Chinese university EFL classrooms based on the data collected from classroom observations and interviews, with an intention to investigate the extent to which teachers engaged their students in assessment and learning and identify teacher factors that influenced the implementation of AfL.

A major finding of our study is that teacher participants adopted different assessment approaches to engage their students. It seemed that Nancy dominated the assessment process, prioritized test preparation, and relied largely on AoL methods to keep students' motivation and efforts in learning English. Luke, although appeared to have got rid of AoL in his classroom, was confined to using mainly closed questions and tasks to engage his students in in-class activities. And as Nancy, Luke's students were not given substantial opportunities to work as assessors in the assessment process. Luke's engagement strategy represents what have been termed convergent assessment (Torrance and Pryor, 1998). Marshall and Drummond (2006) described this type of AfL practice as being implemented to the “letter” because Luke failed to genuinely and fully engage students in learning and assessment. Zack, unlike Luke and Nancy, used open questions strategically to increase his students' cognitive engagement; he also reported implementation of peer- and self-assessment in his previous writing class, which, according to him, had greatly improved students' motivation, confidence, self-efficacy, and learning efforts. Zack's case provided an example of how teachers could approach AfL in a divergent, and “spirit” way to engage students behaviorally, cognitively, and motivationally (Torrance and Pryor, 1998; Marshall and Drummond, 2006).

Our findings also suggest three important teacher factors which appeared to influence teachers' engagement strategies adopted in the assessment process (as summarized in Table 2). First, teachers' assessment literacy, an intrapersonal factor identified by plenty of previous studies of AfL (e.g., Birenbaum et al., 2011; Zhao et al., 2018) see also, Zhang and Zhang, 2020; Harris and Leeming, 2021; Zhang, 2021, for the importance of teachers in the classroom and students' learning process), seemed to influence whether and to what extent teachers used AfL to engage their students in classroom settings. Some researchers argued that teachers need to be assessment literate to ensure high-quality AfL practices, which acknowledge students' agency and empowers them in the assessment process (Heitink et al., 2016; Davison, 2019; Xu, 2019). Assessment literate teachers in relation to AfL are supposed to understand what constitutes quality AfL practices and have adequate knowledge and skills to implement the core AfL strategies (Dixon and Hawe, 2018). For instance, an important aspect of teacher assessment literacy in relation to AfL is the knowledge and skills of constructing and using open questions to engage students in dialogic conversations and discussions; in doing so teachers can elicit abundant in-the-moment information about student deep learning (Erickson, 2007; Ruiz-Primo, 2011; Heritage, 2013). It seemed that Zack, compared with the other two teacher participants, was more skilled in this aspect. Zack was capable of facilitating his students' engagement by asking students to reason and analyze as well as by flexibly reducing and increasing the cognitive demands of his questions on students. Nancy and Luke, however, failed to demonstrate such skills. Luke, for example, although was observed to successfully involved his students in a teacher–student dialogue by using hinge questions, students responses were limited to either “yes/no” or short phrases and they were hence not fully engaged in this dialogue cognitively. Given the fact that Luke and Nancy had limited training in relation to AfL in either their pre- or in-service teacher training courses while Zack had intensive research experience in this field, it seems reasonable to assume that the three teachers had different levels of AfL-related assessment literacy, bringing about different assessment practices to engage their students.

**Table 2:** Summary of the major findings.

	**Assessment literacy**	**Goal orientation**	**Trust**	**Assessment approaches**
Nancy	Lack of assessment literacy	Performance orientation	Mistrust	AoL •In-class examinations •No student-led activities
Luke	Lack of assessment literacy	Learning orientation		Convergent, and “letter” AfL •Close-ended questions •Limited student-led activities
Zack	Assessment literate	Performance and Learning orientations	Mistrust	Divergent and “spirit” AfL in his previous class •Open questions •Substantial student-led activities in previous writing classes but not in his current class

Another influencing factor surfaced from our study was teachers' beliefs about the relationship between goal orientation and motivation, an intrapersonal factor that is rarely discussed in the literature on AfL. Previous theoretical and empirical studies have identified two types of goal orientations: learning orientation and performance orientation (Pintrich, 2000). With a learning orientation, students' purpose in an achievement setting is to develop their understanding and improve their competence and skills. It has been found that students with a learning orientation is characterized by avoidance of not learning, positive affective reaction to failures, and strong motivation and continuous efforts to achieve their goals (Dweck, 1986, 2000; Pintrich, 2000; Dragoni, 2005; Elliot, 2005; Zhang and Zhang, 2018, 2019; Vandewalle et al., 2019). Students with performance orientation, on the contrary, focuses on documenting and demonstrating their abilities and besting others. They usually seek for positive judgments of their competence and try to avoid inferiority. As a result, they may display negative affect and give up their efforts in face of obstacles (Dweck, 2000; Pintrich, 2000; Elliot, 2005). Our study found that all the three teachers perceived that a large percentage of their students held a performance orientation, whose purpose of learning the English courses was to pass examinations instead of improving their English abilities.

However, the three teachers appeared to have differed beliefs about which type of goals might genuinely motivate their students to learn English, and they responded differently to their students' goal orientation, which further influenced their engagement strategies used in classrooms. For example, Nancy believed that advocating a performance orientation and linking the assessment activities to examinations would provide a strong motivation for students to learn the English course, hoping that the high-stakes examinations would compel her students to spend time and effort in learning English. Presumably because of this, Nancy's assessment practices were largely aligned with the AoL tradition; she conducted regular examinations and used bonus scores to ensure student engagement in assessment tasks. In addition, she also refused to implement peer- and self-assessment because she believed her students were more inclined to engage in activities that were closely related with the requirements of examinations. Luck, however, resisted AoL, and his stated reason was that focusing students on the improvement of their English abilities, a learning orientation, was a source of lasting motivation for students to learn English. Likewise, although Zack encouraged some of his students to sign up for examinations, he believed that learning orientation was more helpful in keeping students motivated, interested, and encouraging them to invest effort in English learning; probably for this reason, he also rarely used examinations in his assessment practices. These results send an important message: Teachers should develop a deeper understanding of the role that learning orientations play in engaging students in learning if they are to embrace AfL in their classrooms.

In addition to the previous two intrapersonal teacher factors, our study also affirmed the importance of an interpersonal factor, trust, in the implementation of AfL in Chinese university EFL classes. It seems that our teacher participants lacked competency trust in their students, namely, the confidence that others have the competence and abilities to handle a task (Carless, 2013). This is apparent in Nancy's case, who adopted mainly close-ended tasks, conducted teacher-controlled discourses, and refused to experiment with peer- and self-assessment in her class. This was partly because, as Nancy shared, she doubted her students' abilities to respond to questions with higher cognitive demands as well as their competence to make decisions and judgements about the quality of their work. An interesting finding of our study is that Zack refused to use peer- and self-assessment in his current class although he had realized that this strategy could foster student engagement from his previous experience. As Nancy, Zack's stated reason was that he doubted his current students' language abilities and assessment skills. Moreover, it appeared that he was reluctant to implement peer- and self-assessment in his current class because he also perceived the lack of communication trust among students, which is needed for students to feel psychologically safe to engage in assessment activities, especially student-led ones (Carless, 2013; Xu and Carless, 2017) Nancy and Zack's cases support Carless's (2013) claim that when teachers lack trust in their students, they tend to adopt defensive tasks that leave little space for students to take risks and face challenges, and rely mainly on a transmission teaching approach.

## Conclusions

Adopting a qualitative exploratory case study design, which involved three teachers, our study presented the different assessment practices of three Chinese university EFL teachers in their classrooms. Although AfL has been highly advocated as an effective method to increase student engagement in learning and assessment, not all teachers implemented AfL to the “*spirit*,” which seemed to be a result of a combination of three teacher factors. First, our studies confirm the important role teacher assessment literacy plays in ensuring the effective functioning of AfL (Xu and Brown, 2016; Davison, 2019). Second, we also identify teachers' beliefs about the relationship between students' goals orientation and motivation as an important factor that may influence the way teachers implement AfL to facilitate student learning. Third, consistent with the literature on AfL, our findings also suggest that a trusting relationship between teachers and students was a prerequisite for successful implementation of AfL. These findings indicate that if teachers are to successfully embrace AfL as an approach to engage students, they should be equipped with sufficient AfL-related knowledge and skills, develop a sound understanding of which type of goal orientation can provide an enduring and strong motivation for students in their learning process, and acknowledge their students' agency and invest trust in their students' abilities to take control of their learning. All these points to the significance of the role of teacher education programs (Zhang and Ben Said, 2014; Zhang, 2016, 2021; see Gao and Zhang, 2020; Sun and Zhang, 2021; Yan et al., 2021). We suggest that teacher educators help teachers understand the roles of teachers and students in learning and assessment, provide teachers with clear instructions as to how each specific AfL strategy can be used. In addition, teacher educators may also need to help teachers find effective methods to draw students' attention to the improvement of their abilities, and help teachers start from designing easy tasks to motivate their students to participate in in-class activities and gradually treat their students as partners, and even protagonists, in learning and assessment.

One limitation of our study lies in the small sample size. Only three teachers were recruited in this study, and they were from two universities in Northwest China. Since our participants were chosen on a voluntary basis, maximum variation could not be achieved. More studies focusing on the implementation of AfL in other regions in China are needed to recruit more teacher participates to build up a more complete and clearer picture of how teachers might adopt AfL differently in their classrooms. In addition, as this exploratory study highlights that teachers should be fully prepared for AfL, another area of future research would be to investigate how Chinese teachers' entrenched beliefs in teaching, learning and assessment might change, and how teacher development programs could help teachers acquire and utilize the knowledge and skills needed for the effective functioning of AfL.

## Data Availability Statement

The original contributions generated for the study are included in the article/Supplementary Material, further inquiries can be directed to the corresponding author/s.

## Ethics Statement

The studies involving human participants were reviewed and approved by The University of Auckland Human Ethics Committee. The patients/participants provided their written informed consent to participate in this study.

## Author Contributions

XMW and LJZ conceptualized the research. XMW collected and analyzed the data, and wrote the first draft. LJZ and QL revised the subsequent drafts with XMW. LJZ submitted it as the corresponding author. All authors have made substantial, direct and intellectual contributions to the work, and approved it for publication.

## Funding

This research was based on part of the data for the XMW's Ph.D. research, funded by a joint doctoral scholarship awarded to XMW by The University of Auckland and the China Scholarship Council, Ministry of Education of China.

## Conflict of Interest

The authors declare that the research was conducted in the absence of any commercial or financial relationships that could be construed as a potential conflict of interest.

## Publisher's Note

All claims expressed in this article are solely those of the authors and do not necessarily represent those of their affiliated organizations, or those of the publisher, the editors and the reviewers. Any product that may be evaluated in this article, or claim that may be made by its manufacturer, is not guaranteed or endorsed by the publisher.
