# Supplementary material for: Using Assessment for Learning: Multi-Case Studies of Three Chinese University English as a Foreign Language (EFL) Teachers Engaging Students in Learning and Assessment
Source: Front Psychol. 2021 Sep 30;12:725132. doi: 10.3389/fpsyg.2021.725132 (PMC8515033; doi:10.3389/fpsyg.2021.725132)
Supplement: Supplementary file 1 [file Data_Sheet_1.docx]

**Appendix A**

**Semi-structured Interview Schemes for Teachers**

1. Can you please tell me something about yourself?

2. Can you please talk about your English learning and teaching experience?

3. What do you expect your students to learn in your course?

4. Do you share learning intensions with your students, and how?

5. Do you communicate success criteria with your students, and how?

6. How often do you assess your students’ mastery of knowledge and skills during lessons?

7. Would you please describe or give an example of how you usually assess your students’ mastery of knowledge and skills during lessons?

8. What kind of questions and assessment tasks do you usually use during lessons?

9. Would you please describe or give an example of how you provide feedback do you usually give to your students during lessons?

10. Have you ever given your students opportunities to do self-assessment in this term, and why?

11. Would you please give an example of how you conduct peer-assessment in your current class?

12. What do you do to encourage your students to participate in peer-assessment?

APPENDIX B Example of Qualitative Data Analysis—Zack’s Data

| Themes | Major Pattern Codes | Definition | Examples and Sources |
| --- | --- | --- | --- |
| Course Learning Goals | Perceptions of students’ course learning goals | Teachers’ perceptions of the goals their students aimed to achieve in learning the English course. | “They[study masters] want to learn how to communicate with foreigners in working environment” (Zack Int1) |
|  | Course learning goals advocated | The goals teachers encouraged their students to pursue in learning the English course | “You (teachers) need to encourage them (study slackers) to sign up for CET-4 first” (Zack Int1) |
|  | Reasons for advocating such course learning goals | The reasons why teachers advocated certain goals for their students to pursue in learning the English course | “CET-4 cannot work as a sustained motivation” (Zack Int1) |
| Classroom assessment tasks | Assessment tasks used by teachers | The tasks teachers used to collection information of their students’ learning during lessons. | “Does criticism do more harm than good to people” (Zack Obs1)  “I prefer open-questions” (Zack Int1) |
|  | Reasons for the assessment tasks used | The reasons why certain assessment tasks were used | “[open questions] force my students to expand on their answers rather than merely say a couple of words” (Zack In1) |
| Empowerment of students as assessors | occurrence of peer-and self-assessment | The extent to which teachers implemented peer- and self- assessment during lessons. | “I experimented with [peer- and self -assessment] last term… It was a great succuss” (Zack Int2) |
|  | Reasons for teachers’ peer-and self-assessment practices | The reasons why teachers implement peer-and self- assessment in the way they reported and they were observed | “I can imagine how tiring it will be if I do it (peer-and self-assessment) in my current class. (Zack Int2) |
